# Antifungal and Anticancer Potential of *Argemone mexicana* L.

**DOI:** 10.3390/medicines3040028

**Published:** 2016-11-03

**Authors:** Nilesh V. More, Arun S. Kharat

**Affiliations:** 1 Department of Biotechnology, Dr. Babasaheb Ambedkar Marathwada University, Subcampus, Osmanabad 413501, Maharashtra, India; 2 Department of Biotechnology, College of Computer Science and Information Technology, Latur 413512, Maharashtra, India; nileshm2002@gmail.com

**Keywords:** *Argemone mexicana*, antifungal potential, cold aqueous and methanolic extract, trypan blue assay

## Abstract

**Background:** Medicinal plants are widely used to treat infectious diseases, metabolic disorders and cancer. *Argemone mexicana* L. (*A. mexicana*), commonly found on desolate land of Marathwada (Maharashtra, India) has been used to treat oral cavity infections. **Methods:** In this study, cold aqueous and methanolic extracts were prepared from *A. mexicana* stem and leaves. These extracts were tested for their antifungal and anticancer activities. The antifungal activity was tested using the agar well diffusion method, while the anticancer activity against immortalized cell lines was assessed by trypan blue assay. **Results:** It was observed that both cold aqueous and methanolic extracts of *A. mexicana* stem and leaves inhibited the growth of *Mucor indicus*, *Aspergillus flavus*, *Aspergillus niger* and *Penicillum notatum*. Antifungal activity of the extract was comparable to that of Amphoterecin-B. *A. mexicana* extracts had a cytotoxic effect on A549, SiHa and KB immortalized cell lines that were similar to that of berberine. **Conclusion:** The *A. mexicana* leaf and stems exhibit strong antifungal and anticancer potential.

## 1. Introduction

Ayurveda, the Indian system of natural medicine has been practiced for over two thousand years [1,2]. The response of certain types of infections to antibiotics is poor. To overcome this, some microbial infections like tuberculosis, HIV and hepatitis C virus are treated with a fixed dose drug combination [3,4,5]. Ayurvedic formulations are usually prepared from root, stem, leaf, flower and fruits of medicinal plants. Various infections caused by bacteria, fungi, virus, parasite as well non-infectious metabolic disorders are effectively treated with herbal/Ayurvedic formulations [2,6,7]. There are claims by a number of Ayurveda practitioners that cancer and HIV/AIDS respond well to this type of treatment [7,8,9]. Many commonly used medicinal plants and vegetables not only possess essential nutrients but are also reported to contain secondary metabolites such as alkaloids, flavonoids, glycosides, terpenoids and phenolics. Medicinal plant extracts are beneficial for the maintenance of human health and may be effective in treating chronic degenerative diseases. These compounds also have anti-tumorogenic, immunomodulatory properties and antibacterial potential [10,11,12]. Thus, the identification of the pharmaceutical ingredient from medicinal plants is highly essential [13].

It has been reported that the *A. mexicana* L. (Papaveraceae), commonly known as prickly poppy plant has antimicrobial potential; in particular, in Mexico, Nigeria and tropical America it has been successfully used to treat dental infections [1,10,14]. Alternative medicine practitioners use *A. mexicana* to treat dental infections [1,2]. Fresh yellow, milky seed extract containing protein-dissolving substances is effective for the treatment of warts, cold sores, skin diseases, itching and jaundice [15]. However, there is no report as to whether or not *A. mexicana* can exhibit antifungal activity and act as a control agent of proliferation of infinite cell growth. This work aims to find out whether *A. mexicana* plant has antifungal activity or the ability to slow down massive proliferation of immortalized cell lines in vitro.

## 2. Experimental Section

### 2.1. Materials

#### 2.1.1. Fungal Culture Maintenance

The fungi used in this study included: *Mucor indicus*, *Aspergilus flavus*, *Aspergilus niger* and *Penicillium notatum.* They were maintained on potato dextrose agar (PDA) slants at 4 °C. The bioassay of fungal suspension was obtained by inoculation using potato dextrose broth for 48 h, followed by ten-fold serial dilution in PBS pH 7.2 to obtain CFU/mL = 10^6^.

#### 2.1.2. Cell Culture Maintenance

The human non-small cell lung carcinoma, -A549, human cervical cell, -SiHa, and oral cancer cell, -KB, was obtained from the National Centre for Cell Science (NCCS), Pune, India. They were grown in F-12 HAM’S (A549), MEM-Minimum Essential Medium (SiHA) and DMEM-Dilbecco’s Modified Eagle Medium (KB) culture media, obtained from Invitrogen, Waltham, MA, USA. The culture media was supplemented with heat inactivated fetal bovine serum −10% (Gibco, Invitrogen, Waltham, MA, USA) and antibiotic gentamycin-streptomycin (Gibco, Invitrogen). The in vitro toxicity experiments were carried out on freshly grown monolayer cell lines.

#### 2.1.3. Parts of *A. mexicana* Plant

The plant material was verified and authenticated with the Herbarium Center of Dr. Babasaheb Ambedkar Marathwada University, Aurangabad, India, in the Department of Botany. It was identified as *A. mexicana* L. a member of the Papaveraceae family and is commonly referred to as *Swarnakshiri*, *Bilayat*, *Pivla Dhotra*. The accession number allotted is 0609. Leaves and stems of *A. mexicana* were collected and rinsed with sterile double distilled water, disinfected with 70% alcohol and then dried on paper towel at room temperature. After drying, the plant materials were ground in a laboratory grinding machine.

### 2.2. Methods

#### 2.2.1. Methanolic extracts

In a tightly sealed container at room temperature, fifty grams of grounded plant material was extracted with 150 mL methanol. The extract was protected from light and kept overnight on a rotary shaker, Remi, Elektrotechnik, Ltd., Mumbai, India. The extract was filtered with a five layered sterile muslin cloth. The procedure was repeated three times to obtain clear and colorless filtrate. The methanol from the filtrate was removed by rotary evaporation (Rotary Evaporator, EJER tech, Hangzhou, Zhejiang, China). Extracts were stored at −16 °C overnight and were subsequently freeze-dried at −60 °C in a 20 mL vacuum for 24 h. The extract was then sterilized with UV and stored in an airtight container at 4 °C for further use.

#### 2.2.2. Aqueous Extracts

Fifty grams of grounded plant material was extracted with 150 mL sterile double distilled water for 24 h as in the case of methanol. The mixture was filtered with sterile five-layered muslin cloth and centrifuged at 5000 rpm. The supernatant obtained was concentrated to N/5 volume with rotary evaporator (Rotary Evaporator, EJER tech, Hangzhou, Zhejiang, China). The concentrated extract was then UV sterilized and stored at 4 °C for further use.

### 2.3. Antifungal Potentiality Test

The antifungal potentiality of *A. mexicana* was tested with minor modifications in an agar well diffusion method described by [16]. An inoculum size of 10^6^ CFU/mL was adjusted in molten agar and poured into pre-sterilized petri-plates. Upon solidification, wells were punched with a sterile cork borer (Scientific laboratory, New Delhi, India). Each well was filled with 40 μg extract (20 μL). The Amphoterecin-B (40 μg) was used as the standard for comparison of antifungal activity. Plates were then incubated at 37 °C for 72 h for the detection of inhibitory zone. The experiments were repeated three times, and the average and SD were calculated. Antifungal activity was evaluated by measuring the diameter of growth inhibition zone around the well.

### 2.4. Anticancer Potentiality Test

Independent monolayers in 96 well micro-titration plates were obtained for A549, SiHa and KB immortalized cell lines. The vinblastin served as a positive control while beberine served as an example of alkaloid known to have anticancer potential. Monolayers were inoculated with *A. mexicana* extracts and berberine at concentrations of 50 μg (0.16 μM) up to 300 μg (1 μM) (*w*/*v*) per well. The vinblastin at 20 μg per well was used as a control in this assay. A final volume of 200 μL was adjusted with sterile medium. Plates were then incubated in a CO_2_ incubator for 24 h, 48 h and 72 h. Anticancer potentiality exhibited by *A. mexicana* extract, berberine and vinblastin was estimated by performing a trypan blue cytotoxicity assay as described in Strober [17].

## 3. Results

### 3.1. Antifungal Potential of A. mexicana

**Figure 1:**
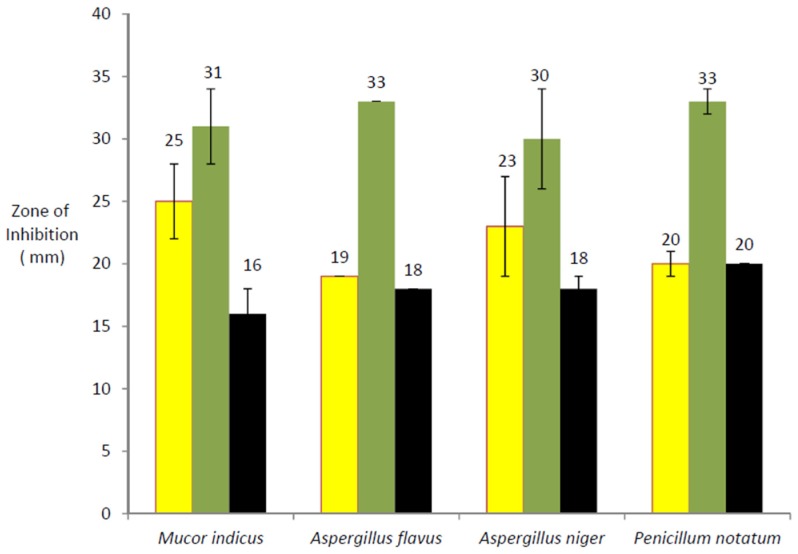
Antifungal activity of methanolic extract against four test fungi: (1) *Mucor indicus*; (2) *Aspergillus flavus*; (3) *Aspergillus niger*; and (4) *Penicillum notatum*. Antifungal activity within methanolic extracts of leaves (yellow bar), stem (green bar) for *A. mexicana* extracts, while black bars indicates antifungal activity of Amphoterecin-B. Error bars shown on each histogram indicates standard deviation.

One of the aims of this study was to address whether or not *A. mexicana* has antifungal potential. The methanolic and cold aqueous extracts prepared from the stem and leaves of *A. mexicana* along with Amphoterecin B were inoculated into wells punched in pre-seeded agar plates. After incubation at 37 °C for 72 h, the clear growth inhibition zone around the well was measured and was recorded as a measure of antifungal activity. The results represented in Figure 1 show that methanolic extracts of *A. mexicana* leaves (yellow bar) for photos (see Supplementary Materials Figure S1) and stems (green bar) exhibit significant antifungal activity for photos (see Supplementary Materials Figure S2). It is clear from Figure 1 that the growth inhibition zone for *M. indicus* with leaf extract was double the size of Amphoterecin-B (black bar). In parallel experiments, growth inhibition zones with stem extracts against all tested fungi were almost double that of Amphoterecin-B. This observation suggests that the antifungal component within the extract could successfully inhibit fungal growth.

**Figure 2:**
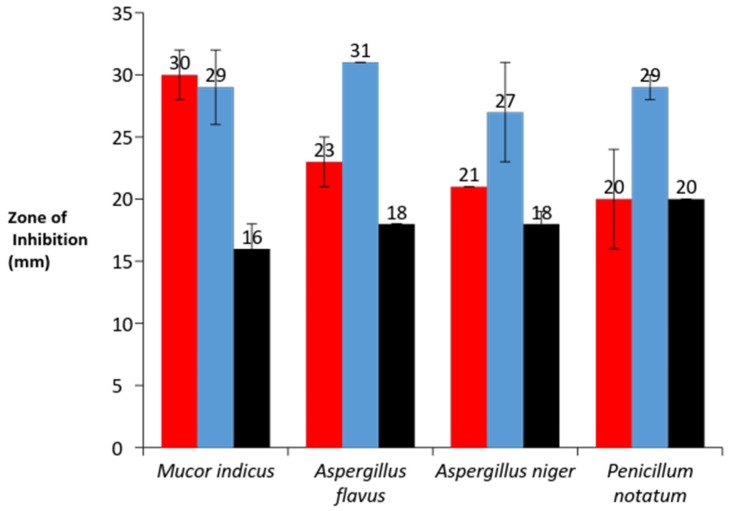
Antifungal potential of *A. mexicana* aqueous extracts: (1) *Mucor indicus*; (2) *Aspergillus flavus*; (3) *Aspergillus niger*; and (4) *Penicillum notatum*. Antifungal activity seen within *A. mexicana* leaf extract (red bar), stem extract (blue bar) while black bars indicate antifungal activity of Amphoterecin-B. Error bars shown on each histogram indicates standard deviation.

It is well known that some organic solutes are more soluble in methanol than in an aqueous base. We sought to find out whether the antifungal compound present in *A. mexicana* stems and leaves was equally soluble both in water and methanol. To address this, cold aqueous extracts from *A. mexicana* stems and leaves were prepared and tested for antifungal potential. Results shown in Figure 2 indicate that inhibitory zones with leaves (red bar) and stems (blue bar) extracts were similar and comparable to that of Amphoterecin B (black bar). Data presented in Figure 1 and Figure 2 demonstrate that *A. mexicana* has antifungal potential, seemingly significant and comparable to that of Amphotericin B.

### 3.2. Anticancer Potential of A. mexicana

**Figure 3:**
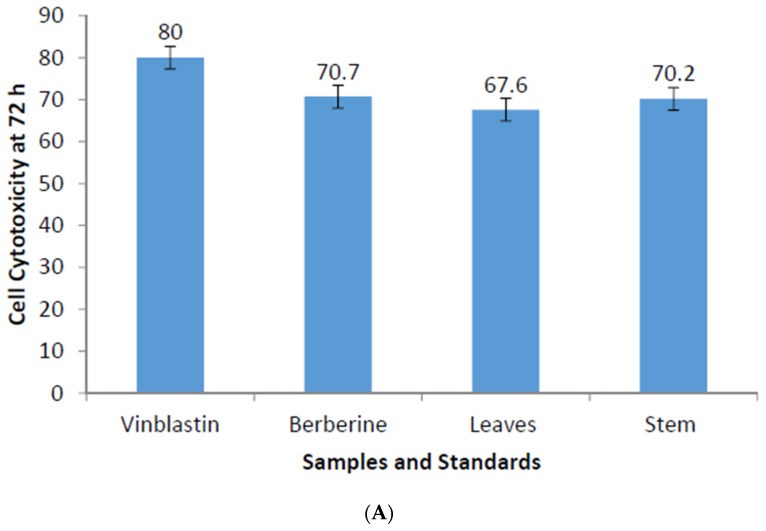

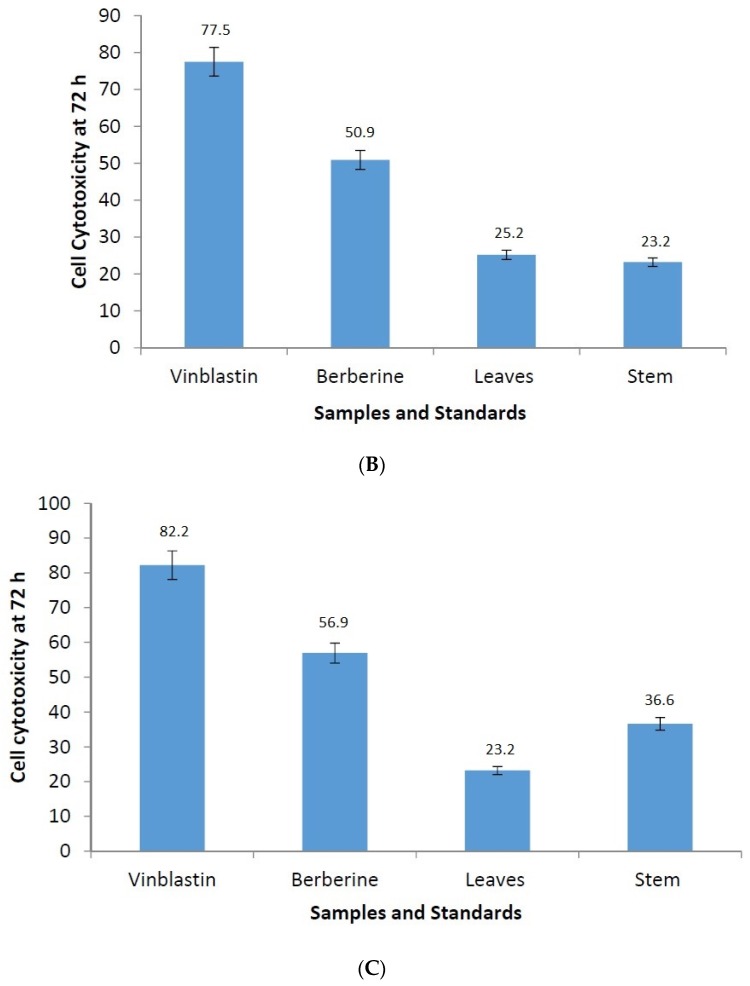
Percentage cytotoxicity of Vinblastine, pure berberine, leaf extracts and stem extracts of *A. mexicana* at 72 h on (**A**) human lung carcinoma cell line -A549; (**B**) cervical cancer -SiHa cell line; and (**C**) oral cancer -KB cell line. Three rounds of experiments were carried out with Vinblastine 20 µg/well, Pure berberine, Leaves and Stem extracts each with 300 µg/well. The bars on each histograms denote standard error.

**Figure 4:**
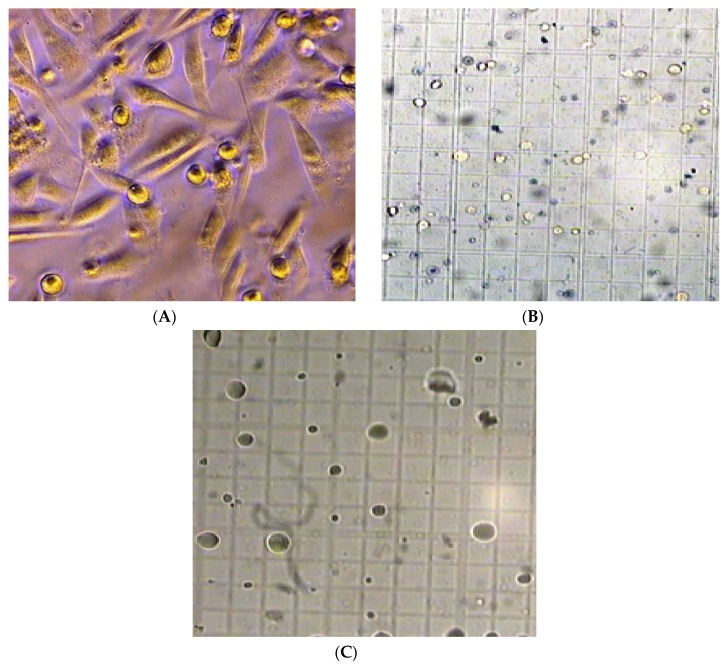
(**A**) Trypan blue dye exclusion staining: the human lung carcinoma cell line -A549 stained with Trypan blue staining immediately upon addition of *A. mexicana* extracts; (**B**) Trypan blue dye exclusion staining: The human lung carcinoma cell line -A549 treated with 300 μg *A. mexicana* leaf extract. Trypan blue staining performed after 72 h of incubation. The black dots denote dead necrotic mass. Hollows and diffused staining denote complete lysis and cell masses in the process of lysis; and (**C**) Trypan blue dye exclusion staining: the human lung carcinoma cell line -A549 treated with 300 μg *A. mexicana* stem extract. Trypan blue staining performed after 72 h of incubation. The black dots denote dead necrotic mass. Hollows and diffused staining denote complete lysis and cell masses in the process of lysis. Images shown used 100× magnification.

After establishing the fact that *A. mexicana* has antifungal potential, we sought to address whether these extracts could exhibit anticancer activity. The cell cytotoxic impact brought by *A. mexicana* extracts was compared with Vinblastin at 20 μg per well (24.5 nM) as a control. Methanolic and cold aqueous extracts of *A. mexicana* leaves and stems were tested over a range of concentrations. An alkaloid—berberine—that has been known to inhibit metmastasis in the SiHA cell line was used at parallel concentrations of *A. mexicana* extracts. Monolayers prepared in 96-well micro-titration plates for immortalized cell lines: A549, SiHa and KB were inoculated with berberine and *A. mexicana* extracts at concentrations of 50 μg to 300 μg per well. The cytotoxic effect on monolayers was estimated with trypan blue assay after 24, 48 and 72 h incubation. Data presented in Figure 3 is an average of three independent experiments, which indicate cytotoxic effect on A549, SiHa and KB immortalized cell lines. The cytotoxic effect seen in the case of A549, 72 h incubation (Figure 3A) with berberine at 300 μg (1μM) per well was 71%, and, with leaf extracts, it was 67%, and with stem extracts, it was 70%. The highest cytotoxicity after 72 h incubation was noticed with vinblastine, which was 82% with 24.5 nM. Parallel experiments were performed with SiHa and KB immortalized cell lines as well (Figure 3B,C). The cytotoxicity exhibited by the *A. mexicana* extracts on the KB cell line was 25% for leaves and 23% for stems, whereas cytotoxicity exhibited in SiHa was 23% with leaf extracts and 36% with stem extracts (shown in Figure 3B,C). Data shown in Supplementary Materials Figure S3A,C,E shows a cytotoxic effect exhibited by standard and *A. mexicana* extracts on A549, SiHa and KB immortalized cell lines after 24 h incubation, whereas Supplementary Materials Figure S3B,D,F shows a cytotoxic effect exerted by standard and *A. mexciana* extracts on A549, SiHa and KB immortalized cell lines after 48 h incubation. Trypan blue staining performed on A549 incubated with *A. mexicana* leaves extract (Figure 4B) and with *A. mexicana* stem extracts (Figure 4C) shows that, after 72 h incubation, most of the cells were dead, showing both necrotic mass as well as cells that are fully lyzed or in the process of lysis. Figure 4A shows trypan blue staining of A549 cells just upon mixing with *A. mexicana* leaf extract, and most of the cells appear to be healthy. These results presented in Figure 3A–C and Supplementary Materials Figure S3A–F demonstrate that the cytotoxicity exhibited by the *A. mexicana* extracts was somewhat comparable to that of an alkaloid—berberine.

## 4. Discussion

In their report, [15,18] demonstrated that oil extracts of *A. mexicana* at various concentrations had inhibitory effects on non-filamentous fungus *Candida albicans*. Our study documents for the first time that aqueous and methanolic extracts prepared from *A. mexicana* L. leaves and stems contain strong antifungal activity against filamentous fungi, namely; *M. indicus*, *A. flavus*, *A. niger* and *P. notatum* (see Figure 1 and Figure 2). The *A. mexicana* seeds, oil extracts and root extracts have been used by tropical medicine practitioners for a long time [1,19]. When contaminated with mustard oil, intoxication of *A. mexicana* results in dropsy. Dropsy cases have been reported in New Delhi, Nepal and other parts of the world [20].

Earlier studies have documented that *A. mexicana* is likely to contain benzylisoquinoline alkaloids such as benzophenanthridines, sanguarine, rotoberberines and protopines, protomexicine, mexitin dehydrocorydalmine, jatrorrhizine, columbamine, dl-tetrahydrocoptisine and dihydrocoptisine [21,22,23,24,25,26]. In an independent study on rodents [22], *A. mexicana* extracts were found to be effective in curing ulcers induced with cystamine. In their work, Zhou et al., [27] used reseveratrol, whereas Mohan et al [28] used ethylgallate to study anti-proliferative effects on human oral squamous immortalized cell line KB. Interestingly, Chu et al., [25] demonstrated that intervention of berberine results in the block of transition from epithelial to mesenchyma. Berberine was also found to inhibit metastasis of the human cervical cancer immortalized cell line SiHa. Studies on anti-proliferation of human lung carcinoma immortalized cell line A549 were done with the use of acriflavine, cucurmin and cucurbitacin B. Use of Curcurbitacin-B caused inhibition of STAT3 pathway and induced apoptosis via oxidative stress and MAP kinase signaling pathway [29,30,31,32].

Albeit with efficacy that is not identical, our results indicate that *A. mexicana* leaves and stem extracts exhibit significant cytotoxicity on A549, SiHa and KB immortalized cell lines. However, cytotoxicity seen with Vinblastine at 24.5 nM was superior over berberine and *A. mexicana* extracts at 300 μg/well. Interestingly, inhibitory activity exhibited by *A. mexicana* extract (see Figure 3) was comparable to that of berberine-HCl. Studies reported by Chu et al., [25] showed that berberine was able to induce reversed epithelium to mesenchymal transition and exhibit inhibitory effects on SiHa. Data presented in this article demonstrates that berberine and *A. mexicana* could induce a cytotoxic effect not only on SiHa but also on KB and A549 immortalized cell lines. The cytotoxicity exerted by the *A. mexicana* extracts on the A549 cell line was better amongst cytotoxicity exerted on the KB and SiHa immortalized cell lines.

## 5. Conclusions

In conclusion, experiments described in this article demonstrate that the *A. mexicana* extracts exhibit both antifungal and anticancer potential. More experiments are required to elucidate molecules that have antifungal/anticancer potential from *A. mexicana* leaves and/or stems.

## Supplementary Materials

The following are available online at www.mdpi.com/2305-6320/3/4/28/s1, Figure S1: Antifungal activity of *A. mexicana* leaves methanolic extract: (**A**) Antifungal activity of leaves against *Aspergillus flavus*; (**B**) *Aspergillus niger*; (**C**) *Penicillum notatum*; (**D**) *Mucor indicus*, Figure S2: Antifungal activity of *A. mexicana* stem methanolic extract: (**A**) Antifungal activity of stem against *Aspergillus flavus*; (**B**)*Aspergillus niger*; (**C**) *Penicillum notatum*; (**D**) *Mucor indicus*, Figure S3: (**A**) Anticancer potentiality of *A. mexicana*: Trypan blue exclusion assay with the use of Vinblastine (24.5 nM), Pure berberine (300 μg), leaves extract (300 μg) and stem extract (300 μg) of *A. mexicana* at 24 h on human lung carcinoma cell line -A549. The histogram indicates % cytotoxicity exhibited by the standard and samples. Data presented is an average of three experiments, the bars on histogram denotes standard deviation; (**B**) Anticancer potentiality of *A. mexicana*: Trypan blue exclusion assay with the use of Vinblastine (24.5 nM), Pure berberine (300 μg), leaves extract (300 μg) and stem extract (300 μg) of *A. mexicana* at 48 h on human lung carcinoma cell line -A549. The histogram indicates % cytotoxicity exhibited by the standard and samples. Data presented is an average of three experiments, the bars on histogram denotes standard deviation; (**C**) Anticancer potentiality of *A. mexicana*: Trypan blue exclusion assay with the use of Vinblastine (24.5 nM), Pure berberine (300 μg), leaves extract (300 μg) and stem extract (300 μg) of *A. mexicana* at 24 h on Crvical cancer cell line -SiHa. The histogram indicates % cytotoxicity exhibited by the standard and samples. Data presented is an average of three experiments, the bars on histogram denotes standard deviation; (**D**) Anticancer potentiality of *A. mexicana*: Trypan blue exclusion assay with the use of Vinblastine (24.5 nM), Pure berberine (300 μg), leaves extract (300 μg) and stem extract (300 μg) of *A. mexicana* at 48 h on Cervical cancer cell line -SiHa. The histogram indicates % cytotoxicity exhibited by the standard and samples. Data presented is an average of three experiments, the bars on histogram denotes standard deviation; (**E**) Anticancer potentiality of *A. mexicana*: Trypan blue exclusion assay with the use of Vinblastine (24.5 nM), Pure berberine (300 μg), leaves extract (300 μg) and stem extract (300 μg) of *A. mexicana* at 24 h on Oral cancer cell line -KB. The histogram indicates % cytotoxicity exhibited by the standard and samples. Data presented is an average of three experiments, the bars on histogram denotes standard deviation; (**F**) Anticancer potentiality of *A. mexicana*: Trypan blue exclusion assay with the use of Vinblastine (24.5 nM), Pure berberine (300 μg), leaves extract (300 μg) and stem extract (300 μg) of *A. mexicana* at 48 h on Oral cancer cell line -KB. The histogram indicates % cytotoxicity exhibited by the standard and samples. Data presented is an average of three experiments, the bars on histogram denotes standard deviation.

## Abbreviations

The following abbreviations are used in this manuscript:

MIC	Minimum Inhibitory Concentration
CFU	Colony Forming Unit
