# Supplementary material for: Antifungal and Anticancer Potential of Argemone mexicana L
Source: Medicines (Basel). 2016 Nov 3;3(4):28. doi: 10.3390/medicines3040028 (PMC5456236; doi:10.3390/medicines3040028)
Supplement: Supplementary file 1 [file medicines-03-00028-s001.pdf]

Supplementary Materials: Antifungal and Anticancer Potential of *Argemone mexicana* L.

Nilesh V. More and Arun S. Kharat

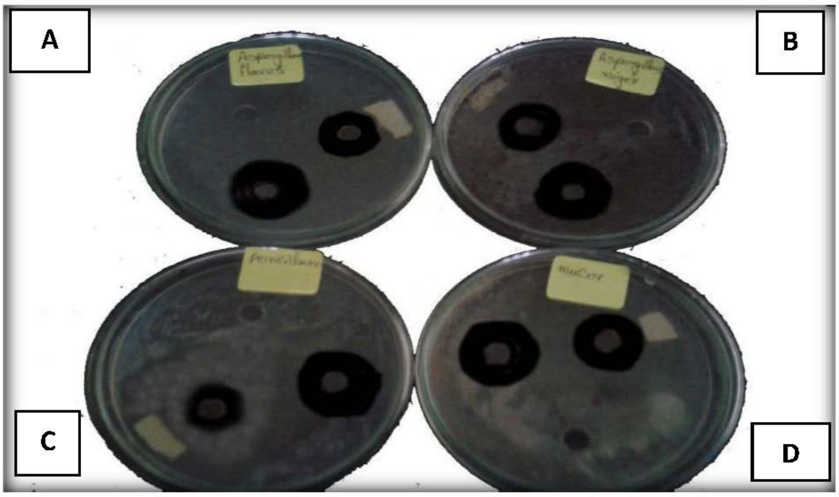

Figure S1. Antifungal activity of *A. mexicana* leaves methanolic extract: (**A**) Antifungal activity of leaves against *Aspergillus flavus*; (**B**) *Aspergillus niger*; (**C**) *Penicillum notatum*; (**D**) *Mucor indicus*.

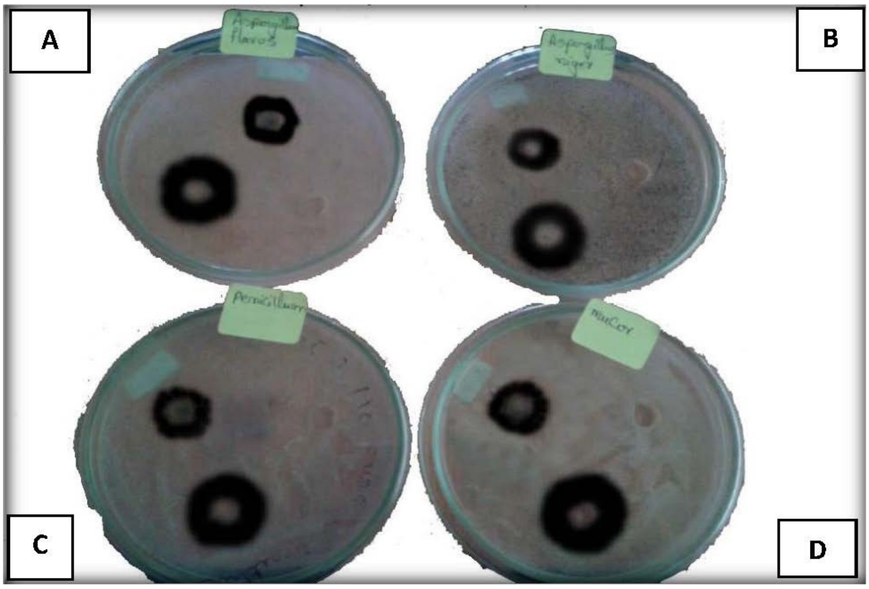

Figure S2. Antifungal activity of *A. mexicana* stem methanolic extract: (**A**) Antifungal activity of stem against *Aspergillus flavus*; (**B**) *Aspergillus niger*; (**C**) *Penicillum notatum*; (**D**) *Mucor indicus*.

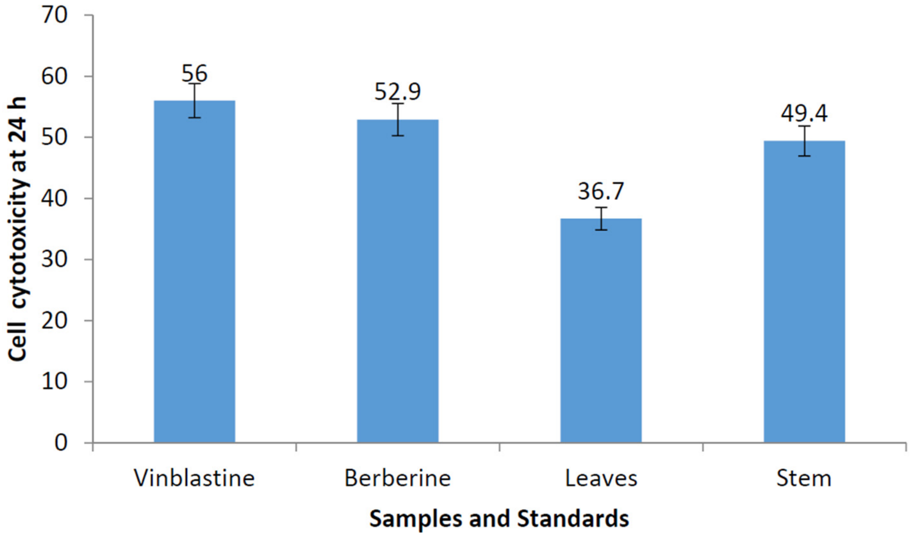

(**A**)

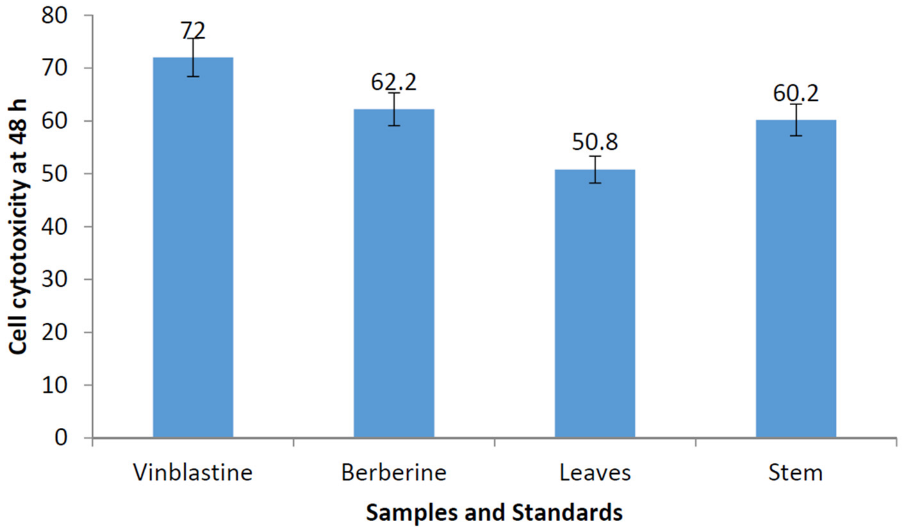

(**B**)

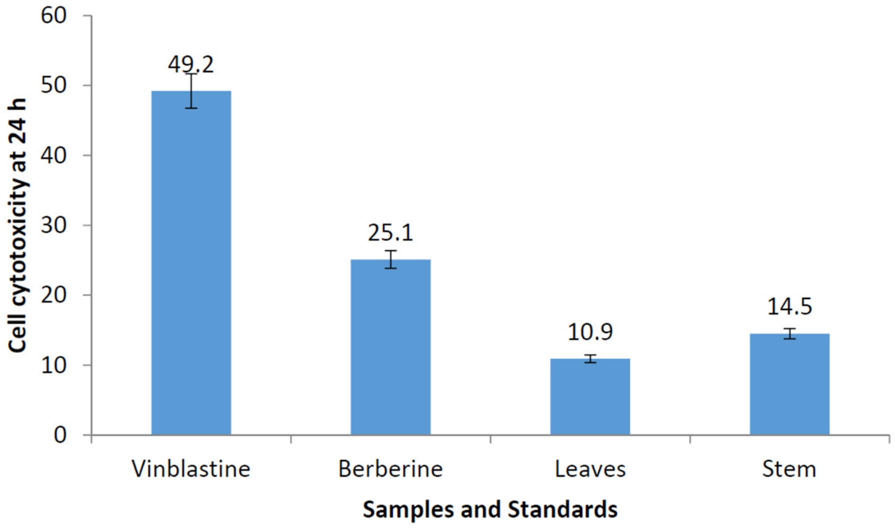

(**C**)

Figure S3. *Cont.*

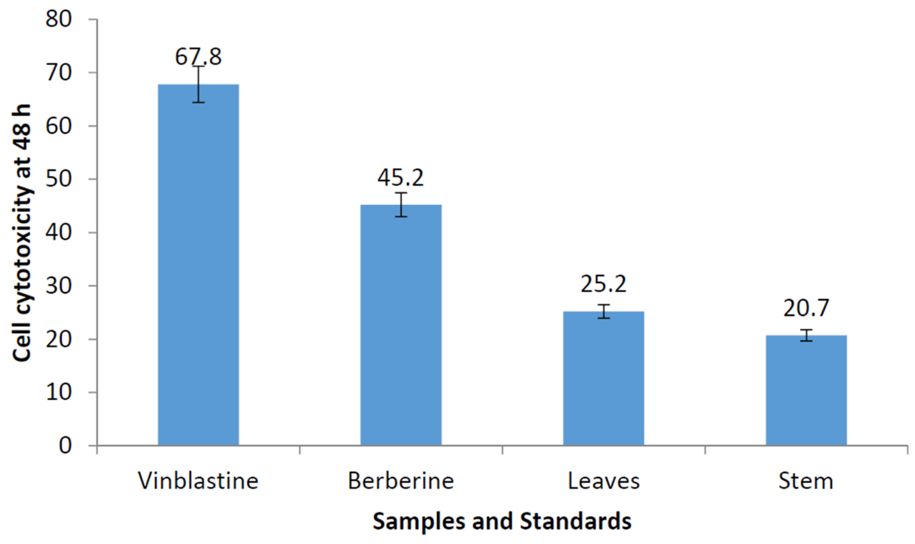
Cell cytotoxicity at 48 h
80
70
60
50
40
30
20
10
0
67.8
45.2
25.2
20.7
Vinblastine
Berberine
Leaves
Stem
Samples and Standards

(**D**)

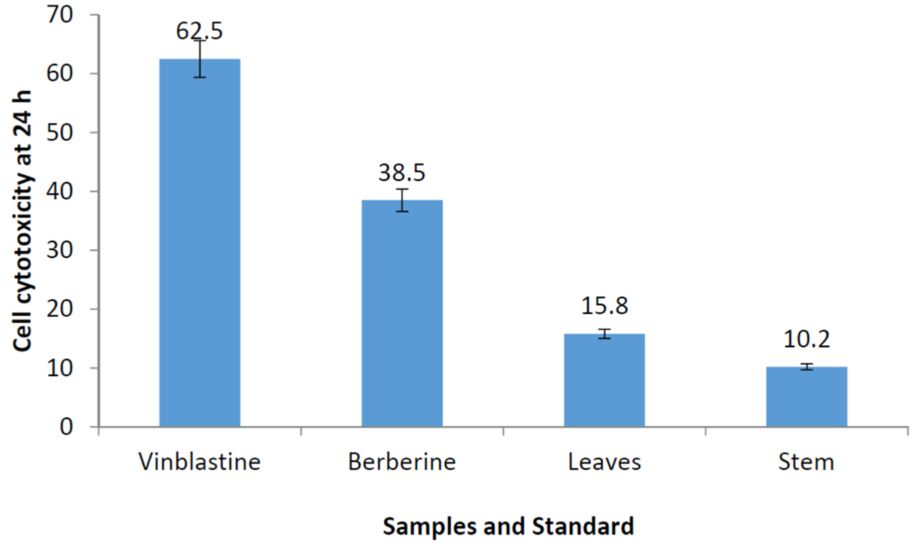
Cell cytotoxicity at 24 h
70
60
50
40
30
20
10
0
62.5
38.5
15.8
10.2
Vinblastine
Berberine
Leaves
Stem
Samples and Standard

(**E**)

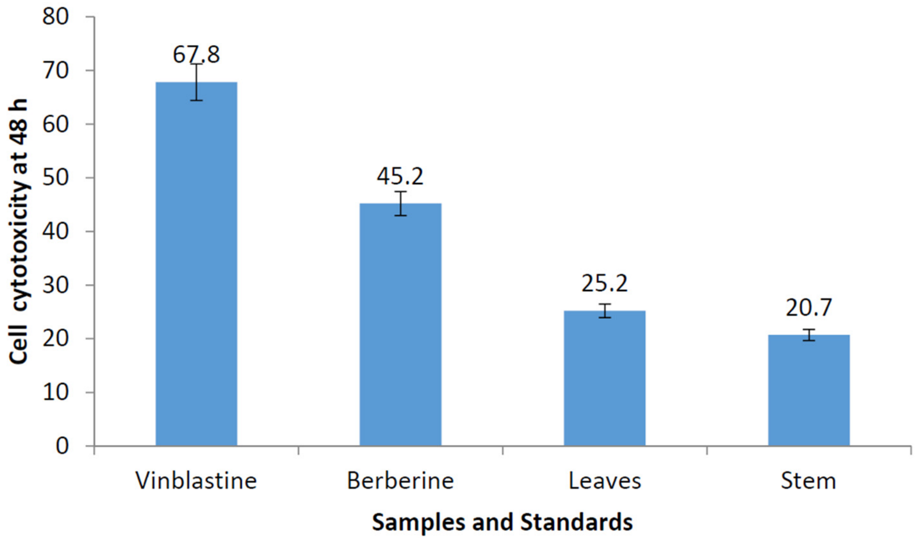
Cell cytotoxicity at 48 h
80
70
60
50
40
30
20
10
0
67.8
45.2
25.2
20.7
Vinblastine
Berberine
Leaves
Stem
Samples and Standards

(**F**)

Figure S3. (**A**) Anticancer potentiality of *A. mexicana*: Trypan blue exclusion assay with the use of Vinblastine (24.5 nM), Pure berberine (300 μg), leaves extract (300 μg) and stem extract (300 μg) of *A. mexicana* at 24 h on human lung carcinoma cell line -A549.The histogram indicates % cytotoxicity exhibited by the standard and samples. Data presented is an average of three experiments, the bars on histogram denotes standard deviation; (**B**) Anticancer potentiality of *A. mexicana*: Trypan blue exclusion assay with the use of Vinblastine (24.5 nM), Pure berberine (300 μg), leaves extract (300 μg) and stem extract (300 μg) of *A. mexicana* at 48 h on human lung carcinoma cell line -A549.The histogram indicates % cytotoxicity exhibited by the standard and samples. Data presented is an average of three experiments, the bars on histogram denotes standard deviation; (**C**): Anticancer potentiality of *A. mexicana*: Trypan blue exclusion assay with the use of Vinblastine (24.5 nM), Pure berberine (300 μg), leaves extract (300 μg) and stem extract (300 μg) of *A. mexicana* at 24 h on Crvical cancer cell line -SiHa. The histogram indicates % cytotoxicity exhibited by the standard and samples. Data presented is an average of three experiments, the bars on histogram denotes standard deviation; (**D**) Anticancer potentiality of *A. mexicana*: Trypan blue exclusion assay with the use of Vinblastine (24.5 nM), Pure berberine (300 μg), leaves extract (300 μg) and stem extract (300 μg) of *A. mexicana* at 48 h on Cervical cancer cell line -SiHa. The histogram indicates % cytotoxicity exhibited by the standard and samples. Data presented is an average of three experiments, the bars on histogram denotes standard deviation; (**E**) Anticancer potentiality of *A. mexicana*: Trypan blue exclusion assay with the use of Vinblastine (24.5 nM), Pure berberine (300 μg), leaves extract (300 μg) and stem extract (300 μg) of *A. mexicana*at 24 h on Oral cancer cell line -KB. The histogram indicates % cytotoxicity exhibited by the standard and samples. Data presented is an average of three experiments, the bars on histogram denotes standard deviation; (**F**) Anticancer potentiality of *A. mexicana*: Trypan blue exclusion assay with the use of Vinblastine (24.5 nM), Pure berberine (300 μg), leaves extract (300 μg) and stem extract (300 μg) of *A. mexicana* at 48 h on Oral cancer cell line -KB. The histogram indicates % cytotoxicity exhibited by the standard and samples. Data presented is an average of three experiments, the bars on histogram denotes standard deviation.
